# The Mechanisms of Carnosol in Chemoprevention of Ultraviolet B-Light-Induced Non-Melanoma Skin Cancer Formation

**DOI:** 10.1038/s41598-018-22029-x

**Published:** 2018-02-23

**Authors:** Lingying Tong, Shiyong Wu

**Affiliations:** 1 Edison Biotechnology Institute, Ohio University, Athens, Ohio 45701 USA; 2 Department of Chemistry and Biochemistry, Ohio University, Athens, Ohio 45701 USA

## Abstract

Carnosol is a natural compound extracted from rosemary and sage, which has been demonstrated to have anti-inflammatory, anti-oxidant, and anti-cancer properties. In this report, we evaluated the therapeutic potential and elucidated the potential mechanism of action of carnosol in chemoprevention of ultraviolet B-light (UVB) induced non-melanoma skin cancer formation. Our data indicated that carnosol could partially reduce UVB-induced reactive oxygen species (ROS) elevation and thus reduce DNA damage. It could also reduce UVB-induced formation of cyclobutane pyrimidine dimers (CDP) in keratinocytes possibly through its ability in absorbing UVB radiation. In addition, carnosol could inhibit the UVB-induced activation of NF-κB and also reduce UVB-induced transformation of keratinocytes. Taken together, the results indicate the role of carnosol as a potential chemopreventive agent upon UVB radiation.

## Introduction

**Figure 1:**
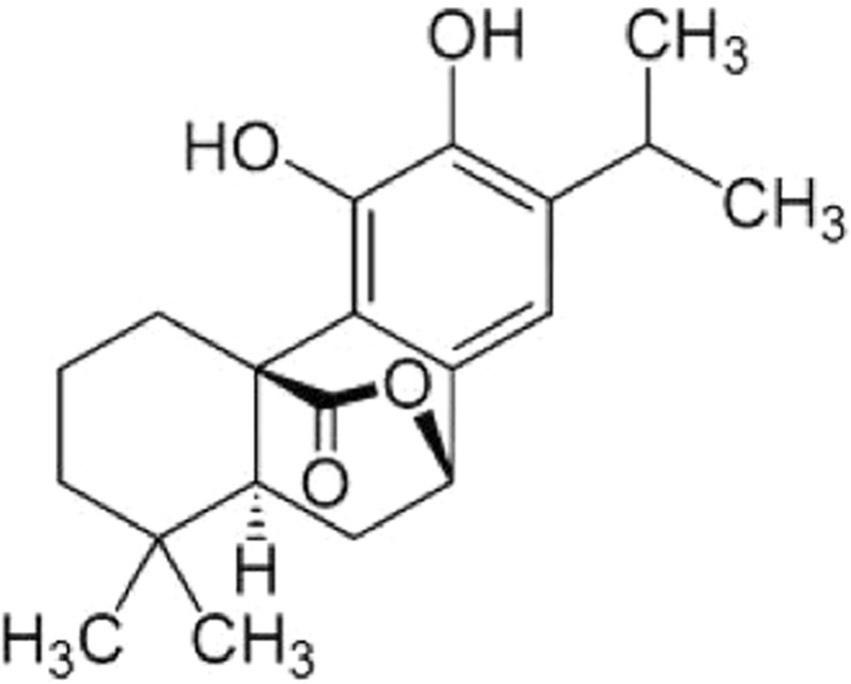
The chemical structure of carnosol.

Carnosol is a natural compound extracted mainly from rosemary and sage, which are both common ingredients used in traditional Mediterranean cuisine^1^. Mediterranean diet and herbs are known to be associated with decreased risks of cardiovascular and diabetic diseases for decades^2,3^. Most recently, the identification and characterization of the anti-cancer properties of these herbs have received intensive interests^4,5^. Among all the compounds extracted from these herbs, carnosol (Fig. 1), first isolated from sage in 1941, has been demonstrated to have anti-inflammation, anti-oxidation and anti-cancer properties^6–8^. Due to its structure similarity to sex hormones, carnosol has been shown to inhibit the growth of prostate and breast cancers by binding to estrogen and androgen receptors respectively^9–13^. However, no study has been done so far on the chemopreventive potential of carnosol on skin cancer upon Ultraviolet B (UVB) radiation. There are two reasons that carnosol can be a good candidate for chemoprevention of skin cancer formation: one is that it has an absorbance peak at 284 nm^14^, which overlaps the wavelength of UVB, a well-known environmental carcinogen that causes various skin cancer^15,16^; and the other is that it has the ability to scavenge reactive oxygen species (ROS)^17^, which is known to be involved in carcinogenic mechanisms upon UVB radiation^18^. In this study, we studied the functions of carnosol in regulating UVB-induced ROS elevation and DNA damage as well as cell carcinogenesis. We provided evidences that carnosol could potentially be a therapeutic agent for chemoprevention of UVB-induced skin cancers.

## Results

### Carnosol reduces UVB-induced ROS in human keratinocytes

**Figure 2:**
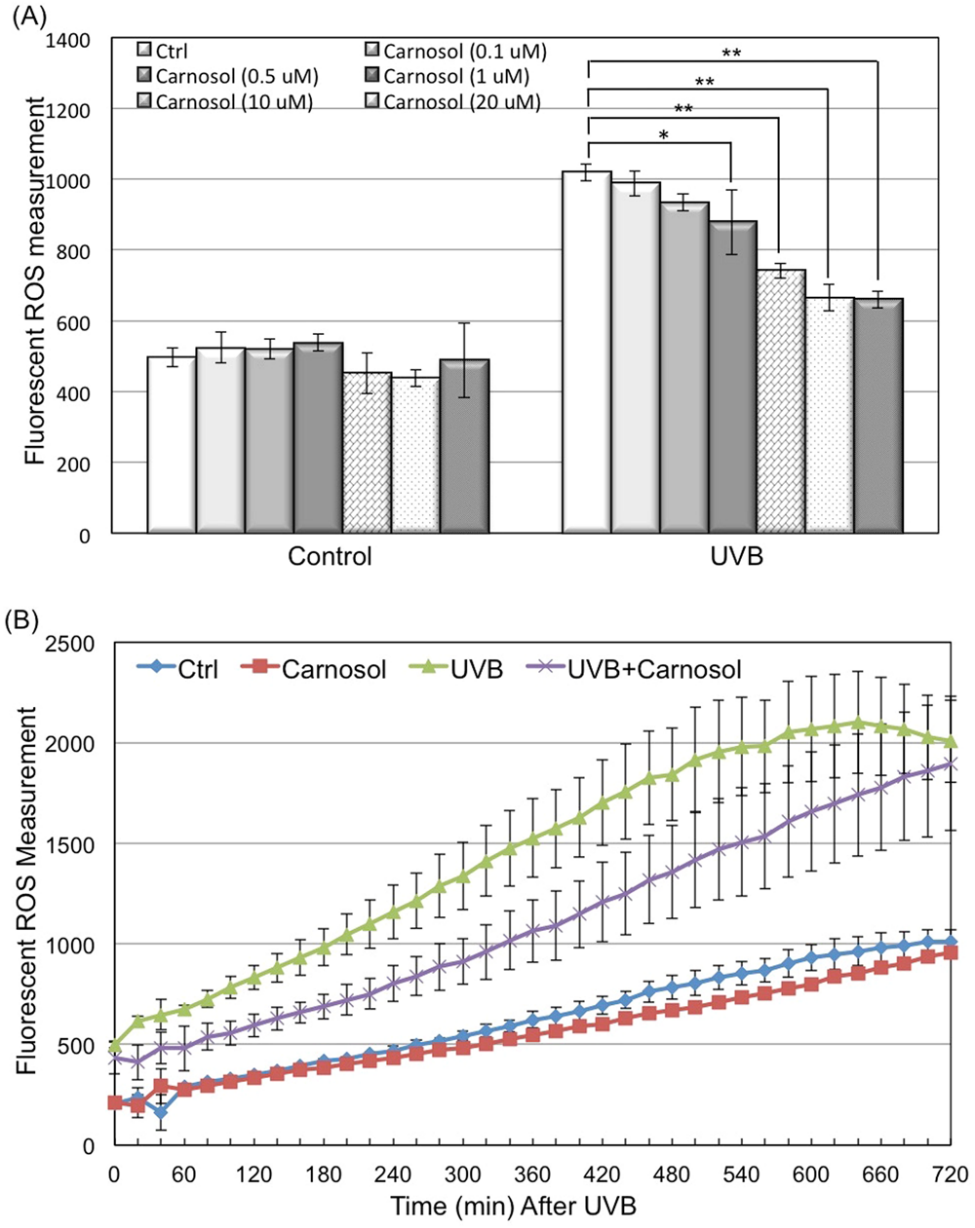
Dose and time dependent effect of carnosol on UVB-induced intracellular ROS level. HaCaT cells were seeded in 96 well plate and incubated with CM-H_2_DCFDA dye 60 minutes prior to UVB exposure, with or without carnosol treatment. (**A**) ROS was measured at 6 hours post UVB radiation with indicated concentration of carnosol treatment using ex490/em520 nm. (**B**) Carnosol (20 μM) was added to cells 60 minutes prior to UVB exposure and ROS level was measured at 20 minutes intervals post UVB radiation until 12 hours. *p < 0.05, **p < 0.01.

We first determined the effect of different doses of carnosol on intracellular ROS level in HaCaT cells with or without UVB radiation. Our data showed that carnosol treatment alone (0.1 μM to 30 μM) had no statistically significant effect on the background ROS level in cells. UVB (50 mJ/cm^2^) radiation caused 2-fold ROS induction at 6 hours after radiation (Fig. 2A). Carnosol treatment at 0.1 μM or 0.5 μM had no statistically significantly change at ROS level post-UVB; however, ROS levels were decreased to approximately 1.5-fold by 10 μM carnosol treatment and 1.3-fold by 20 or 30 μM carnosol treatment. The results indicated that carnosol reduced UVB-induced ROS level in a dose-dependent manner. Since 30 μM carnosol showed no further deduction on ROS level compared to 20 μM, we used 20 μM carnosol treatment for further analysis (Fig. 2A).

After selecting the dose of carnosol, we next determined the effect of carnosol on UVB-induced ROS elevation in a time dependent manner. We measured the ROS level at 20 minutes intervals for 12 h post UVB radiation. Our data indicated that carnosol treatment did not statistically significantly affect the ROS level in non-radiated cells, but it continuously reduced the ROS level in the irradiated cells from 20 minutes to 10 h after UVB radiation (Fig. 2B). These results indicated that carnosol might selectively inhibit the induction of some ROS induced by UVB radiation.

### Carnosol protects DNA from UVB-induced breakage

**Figure 3:**
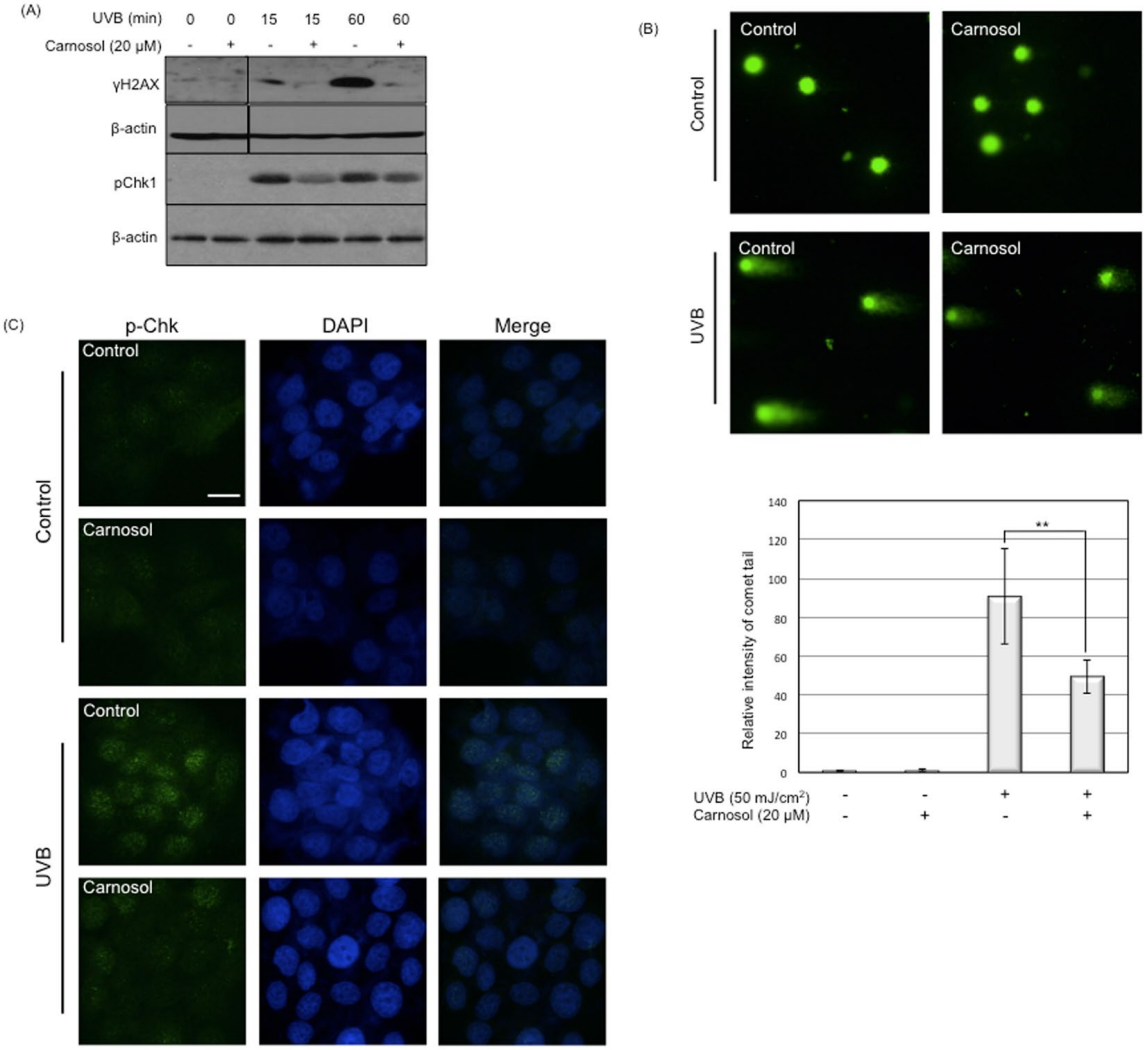
Carnosol protects UVB-induced DNA damage. HaCaT cells were exposed to 50 mJ/cm^2^ UVB radiation in the presence or absence of carnosol (20 μM). (**A**) Cells were lysed at indicated time point and protein levels of phosphorylated H2AX (γH2AX) and Chk1 (p-chk1) were determined by western blot. The data represents three sets of independent experiments. (**B**) Comet assay for HaCaT exposed to UVB radiation. After cell treatment, cells were trypsinized and collected for comet assay. The tail intensity was semi-quantitatively analyzed using Image J. *p < 0.05, **p < 0.01. (**C**) Cells were immunostained with p-Chk1 upon UVB radiation with or without carnosol (20 μM) treatment. p-Chk1 was stained with green fluorescent dye, while nucleus was stained with DAPI. Scale indicates 20 μm.

As increased levels of intracellular ROS causes DNA damage^19^, we next determined whether the reduced ROS by carnosol is correlated to DNA damage upon UVB radiation. We used phosphorylated H2AX (γH2AX) and Chk1 (p-Chk1) as two DNA breakage and damage markers^20,21^. In HaCaT cells, at early phase (15 and 60 minutes) post UVB radiation, the phosphorylation levels of both H2AX and Chk1 was significantly reduced by carnosol treatment (Fig. 3A). We further confirmed the DNA damage in cells using comet and immunofluoresent assays. In comet assay, the intensity of the comet tail was reduced by almost 50% comparing the cells treated with carnosol (20 μM) and non-treated ones (Fig. 3B). The immunofluorescent assay showed decreased level of p-Chk1 in the nucleus when treated with carnosol (20 μM) in UVB irradiated HaCaT cells (Fig. 3C). These results indicated that carnosol could at least partially protect the DNA breakage from UVB radiation.

### Carnosol reduces UVB-induced DNA lesions formation

**Figure 4:**
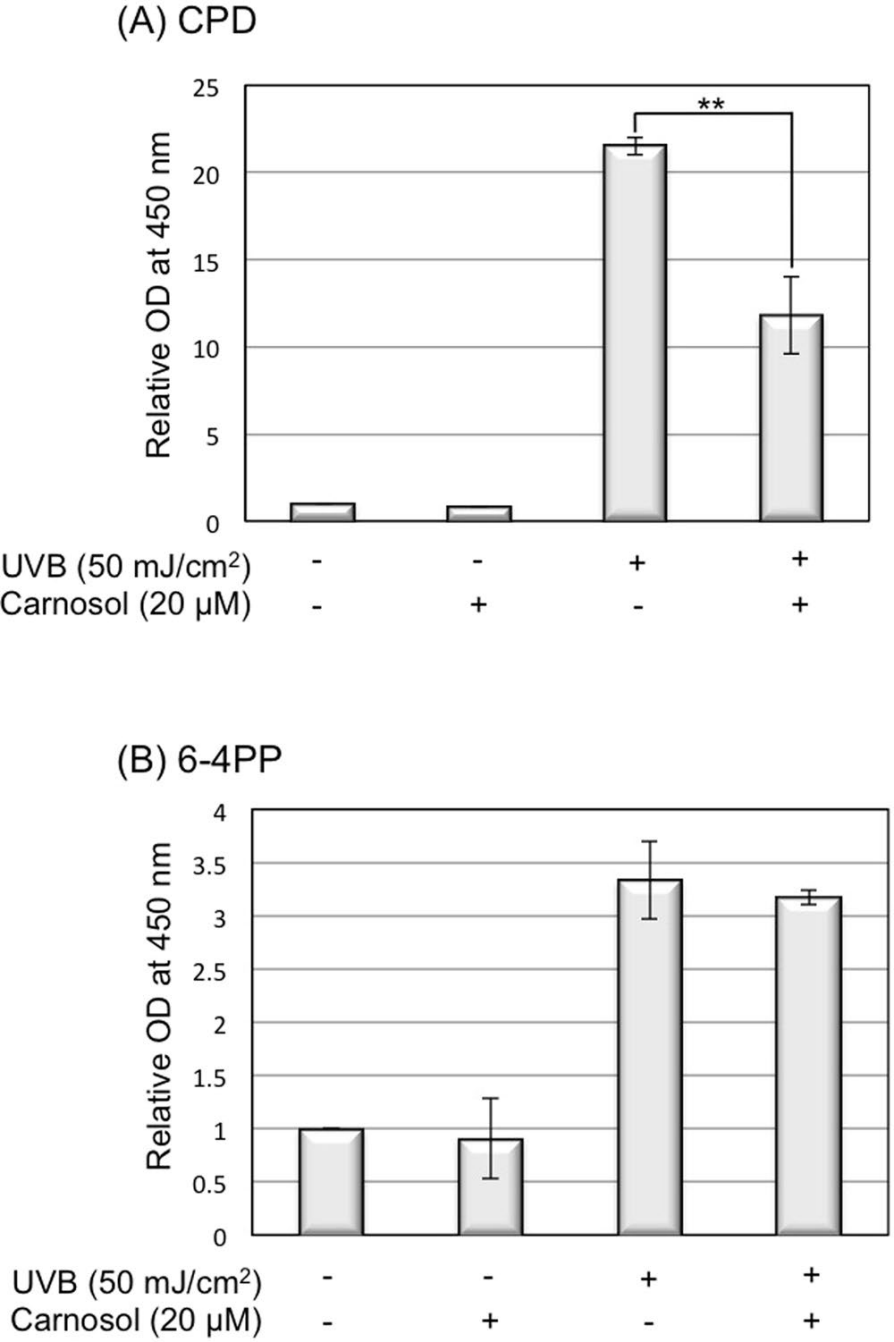
Carnosol reduces the formation of CPD upon UVB radiation. HaCaT cells (with or without carnosol treatment) were collected 15 minutes after 50 mJ/cm^2^ UVB radiation. DNA was extracted from the cells and the production of CPD and 6–4 PP were detected by ELISA assay. (**A**) CPD quantitative detection. (**B**) 6–4 PP quantitative detection. **p < 0.01.

UVB can also damage DNA by inducing the formation of DNA lesions^22^. The two predominant forms of UVB-induced DNA lesions are cyclobutane pyrimidine dimers (CPD) and 6–4 pyrimidone photoproducts (6–4 PP)^23^. To determine whether the formation of DNA lesions is also protected by carnosol, we quantitatively analyzed the UVB-induced formation of CPD and 6–4 PP for cells treated with or without carnosol. Our data demonstrated that UVB radiation increased CPD and 6–4 PP formations by 21.5-fold and 3.3-fold, respectively. Carnosol treatment could decrease UVB-induced CPD formation by approximately 50% to 11.8-fold (Fig. 4A); while the treatment has no significant effect on UVB-induced 6–4 PP formation (Fig. 4B). These results indicated that carnosol might be able to compete with DNA in absorption of UVB radiation.

### Carnosol inhibits keratinocyte transformation upon UVB radiation

**Figure 5:**
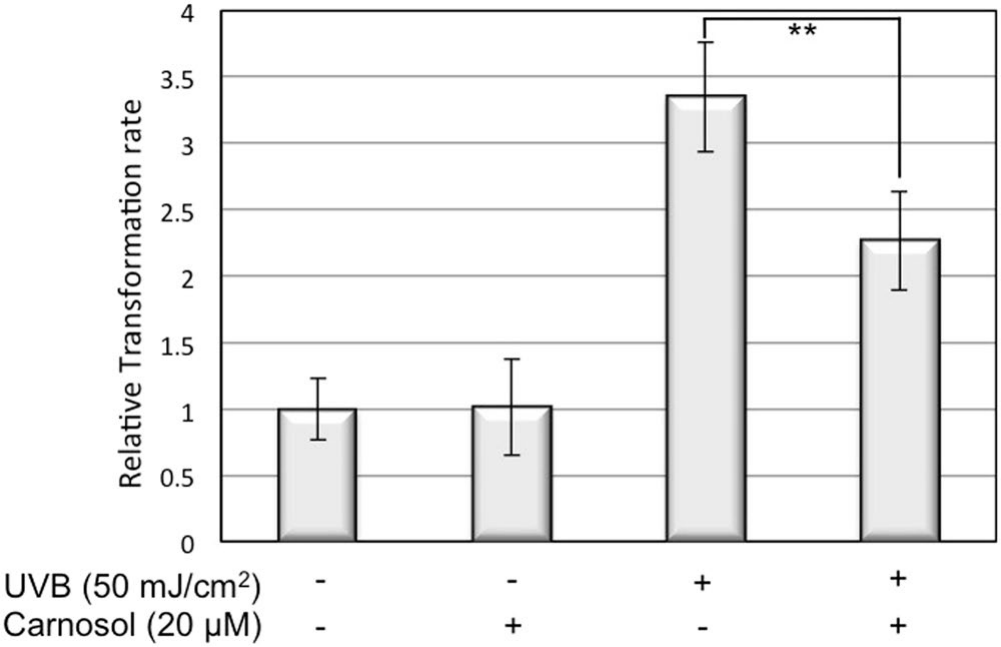
Carnosol decreases HaCaT transformation rate upon UVB radiation. HaCaT cells were exposed to 10 mJ/cm^2^ UVB radiation every 48 hours for 14 days. Cells were then collected and equal number of cells was re-seeded into 96-well plate with soft agar. After 10 days of incubation, cells were stained and the fluorescent intensity was determined. **p < 0.01.

UVB-induced DNA breakages and lesions are known to be one of the major risk factors in cancer development^24^. To evaluate the potential chemopreventive effect of carnosol on UVB-induced skin cancer formation, we determined the transformation rate of keratinocytes in the presence of carnosol after repeated UVB radiation using soft agar assay. Our data demonstrated that the treatment of carnosol reduced the UVB-induced transformation of keratinocytes from 3.5-fold to 2.2-fold, which is an approximately 50% reduction. This result indicated that carnosol could protect skin cells from transforming to cancerous cell upon UVB radiation (Fig. 5).

### Carnosol helps cell survive upon UVB radiation

**Figure 6:**
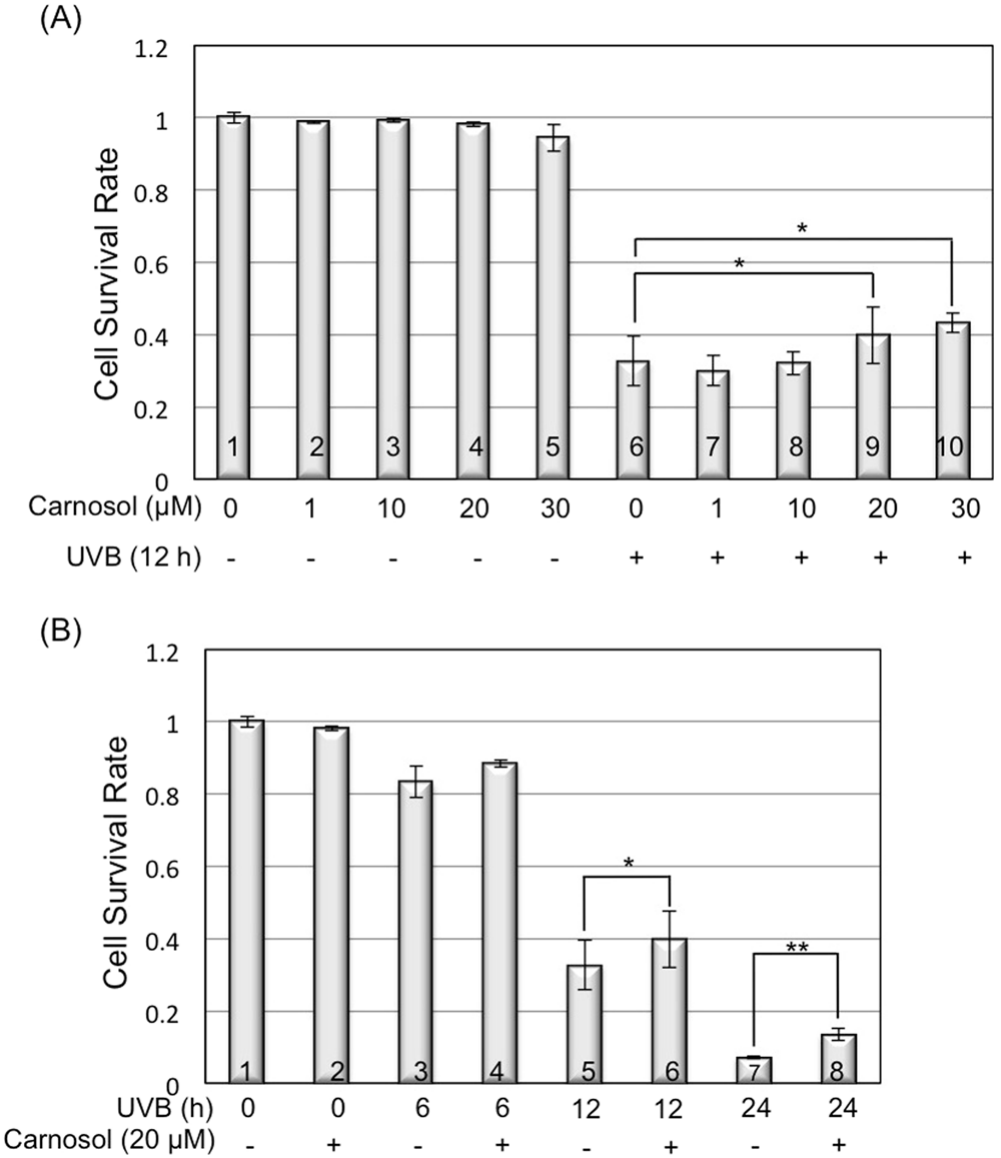
Carnosol protects UVB-induced cell death. Cells were exposed to UVB radiation with different doses of carnosol treatment. At indicated time point, cells were collected and Annexin V/PI apoptosis detection kit was used to detect cell apoptosis. (**A**) HaCaT cell survival rate with different concentrations of carnosol treatment at 12 hours post UVB radiation. (**B**) Time dependent assay in the presence or absence of carnosol treatment in HaCaT cells. *p < 0.05, **p < 0.01.

Because carnosol reduced intracellular ROS level and protected cells from UVB-induced DNA damage and cell transformation, we further examined if it could maintain the cell survival rate after UVB radiation. The dose-dependent analysis revealed that carnosol alone (1–30 μM) had no statistically significant effect on cell survival (Fig. 6A, Lanes 2–5 vs. 1). With UVB radiation, carnosol had no statistically significant effect on cell survival at concentrations of 1 and 10 μM (Fig. 6A, Lanes 7,8 vs. 6); while increased cell survival rate from 30% of UVB radiation alone to approximately 40% at concentrations of 20 and 30 μM (Fig. 6A, Lanes 9,10 vs. 6). The time-dependent analysis revealed that carnosol (20 μM) treatment increased cell survival by 5.2% (not statistically significant), 7.2% and 6.2% at 6, 12 and 24 h post UVB irradiation (Fig. 6B, Lanes 4,6,8 vs. 3,5,7). These results indicated that carnosol had a slight protective effect on UVB-induced cell death.

### Carnosol inhibits UVB-induced NF-κB activity

**Figure 7:**
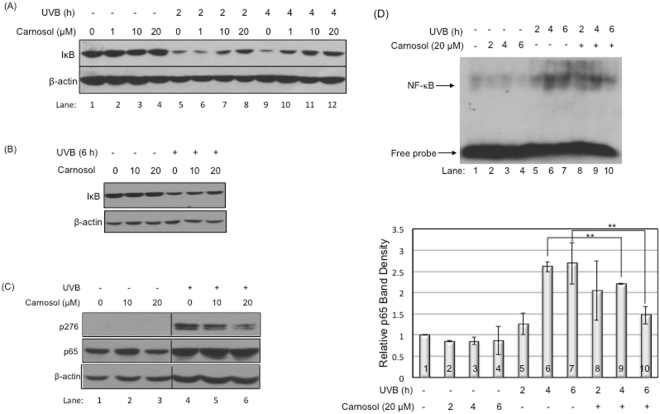
Carnosol inhibited UVB-induced NF-κB activation. Cells were exposed to UVB radiation (50 mJ/cm^2^) in the presence or absence of carnosol. Cells were lysed at indicated time point and protein levels were determined by western blot analysis. (**A**) Western blot for IκB protein level in HaCaT cells at 0, 2, 4 hours post UVB radiation at 1, 10 or 20 μM carnosol treatment. (**B**) Western blot analysis for IκB protein level in HaCaT cells at 6 hours post UVB radiation in the presence or absence of carnosol (10 or 20 μM). (**C**) Western blot for S276 site phosphorylation of NF-κB in HaCaT cells at 6 hours post UVB radiation with 10 or 20 μM carnosol treatment. See supplement material for full-length western blot figure. (**D**) EMSA assay and its statistical analysis for HaCaT cells at 0, 2, 4, 6 hours post UVB radiation in the presence or absence of carnosol (20 μM). **p < 0.01.

Since the protective effect of carnosol on UVB-induced cell death is not as significant as we predicted, we further determined if carnosol could suppress UVB-induced activation of the pro-survival NF-κB pathway as we previously reported^25^. Our data showed that carnosol could partially protect IκB, the inhibitor of NF-κB, from UVB-induced reduction in a dose-dependent manner (1, 10, 20 μM) (Fig. 7A Lanes 6–8 vs. 5; Lanes 10–12 vs. 9). The protection lasted at least 6 hours post-UVB radiation (Fig. 7B). The increased levels of IκB were correlated to a decreased phosphorylation of NF-κB at S276 at 20 μM concentration (Fig. 7C, Lanes 5,6 vs. 4). The NF-κB activity was further confirmed by electrophoretic mobility shift assay (EMSA). By UVB alone, the DNA-binding activity of NF-κB increased about 20% at 2 hours (Fig. 7D Lane 5 vs. 1) and peaked to approximate 2.6-fold at 4 and 6 hours (Fig. 7D Lanes 6,7 vs. 1) post-UVB irradiation. While the treatment of carnosol (20 μM) did not have a statistically significant effect at 2 hours post-UVB irradiation (Fig. 7D Lane 8 vs. 5), the treatment reduced the UVB-induced NF-κB activity from its peak 2.6-fold to 2.2- and 1.5-fold at 4 and 6 hours post-UVB irradiation respectively (Fig. 7D, lane 9 vs. 6 and lane 10 vs. 7). These results indicated that carnosol could inhibit UVB-induced activation of NF-κB, which is often considered as a pro-survival and pro-oncogenic factor^26^.

## Discussion

Overexposure to UVB leads to an increased chance of developing various forms of skin cancers, including basal cell carcinoma (BCC), squamous cell carcinoma (SCC) and cutaneous malignant melanoma^15,16^. Identification and characterization of natural compounds as chemopreventive agents for UVB-induced skin cancer formation is very appealing because these compounds are often safer and more environmentally friendly. In this study, we determined the efficacy of carnosol, a natural compound extracted from sage or rosemary, in protecting keratinocytes from UVB-induced DNA damage and transformation. Our results indicated that carnosol reduced skin cell transformation (Fig. 5) possibly through the following mechanisms. First, carnosol protects DNA from UVB-induced breakage by reducing intracellular ROS level in the irradiated cells (Figs 2 and 3). Second, carnosol reduces DNA lesion by reducing the formation of CPD (Fig. 4A), but not 6–4 PP (Fig. 4B)^27^. The selective reduction could be due to that the absorbance wavelength for forming cis-syn CPD is at 280 nm^28^, which overlaps with the absorbance peak of carnosol in 284 nm^14^; on the other hand, the absorbance wavelength to form 6–4 PP and its Dewar valence isomers is at 313 nm^28^ which has no overlap with the absorbance of carnosol. This makes carnosol a good candidate for chemoprevention of UVB-induced skin cancer because (1) CPD is the predominant form of the dTpT dimers induced by UVB radiation (Fig. 4, Panel A vs. B)^30^ and (2) the lasting UVB-induced mutation is caused by CPD but not 6–4 PP or oxidative DNA damage^29^. Moreover, carnosol inhibits UVB-induced NF-κB activation (Fig. 7), which is a pro-oncogenic factor^26^ elevated in skin cancer cells^31^. Thus, the inhibition of NF-κB activity could potentially reduce the risk of cancer development after overexposure to UVB radiation. Carnosol, a polyphenol, may inhibit UVB-induced NF-κB activation through NADPH oxidase (NOX)^32^, which is known to be activated by UVB and products superoxide (O_2_^•−^)^33^. We previously reported that the UVB-induced early activation of NF-κB is dependent on constitutive nitric oxide synthases (cNOS) activation^25^. The nitric oxide (NO^•^) produced from cNOS can quickly react with O_2_^•−^ to form peroxynitrite (ONOO^−^), which leads to PERK activation, eIF2α phosphorylation, down regulation of IκB synthesis and sequentially NF-κB activation^25,34–36^. By inhibiting NOX and reducing the production superoxide (O_2_^•−^), carnosol could reduce the formation of peroxynitrite (ONOO^−^), thus down regulate NF-κB activation. Based on these results, carnosol shows the characteristics to be a chemopreventive agent upon UVB-induced skin cancer.

## Material and Methods

### Cell Culture and drug treatment

Human keratinocyte HaCaT cells (kindly provided by Dr. Nihal Ahmad, University of Wisconsin-Madison) were grown in Dulbecco’s Minimal Essential Medium (Cellgro) supplemented with 10% fetal bovine serum and penicillin/streptomycin, at 37 °C with 5% CO_2_. Carnosol (Cayman) was added to cells at indicated concentration at 60 minutes before UVB radiation. After radiation, cells were continuously incubated with or without carnosol in the medium until further analysis.

### UVB Radiation

UVB was generated from a Bench XX-Series UV Lamp (UVP Inc.) equipped with two 15-watt UVB tubes (UVP Inc.). The intensity of UVB was calibrated by a UVP model UVX digital radiometer (UVP Inc.) after 5 minutes warming up of the lamps. The dose rate for 10 mJ/cm^2^ or 50 mJ/cm^2^ of UVB radiation was 0.8 or 3.8 mW/s respectively. Medium was removed before exposing the cells to UVB. After UVB radiation, fresh medium was added to the culture plates with or without drugs, and the cells were kept in incubation at 37 °C with 5% CO_2_ until further analysis.

### ROS measurement

CM-H_2_DCFDA (Invitrogen) was used to measure the total ROS level in cells. CM-H_2_DCFDA was dissolved in DMSO to a stock solution of 500 μM and diluted in PBS to final concentration of 5 μM. CM-H_2_DCFDA was added into cells 60 minutes prior to UVB exposure and the reading of fluorescence dye was recorded every 20 minutes using luminometer (Molecular devices Spectra Max M2).

### Cell transformation assay

HaCaT cells were radiated by 10 mJ/cm^2^ UVB every 48 hours for 14 days with or without carnosol (20 μM) treatment. Transformation assay was performed following the protocol of 96-well cell transformation assay (Cell Biolabs, Inc.). Base agar layers were prepared using 1.2% agar solution with 2X DMEM/20% FBS, and solidified at 4 °C for 30 minutes. Cell agar layers were prepared similarly and 5000 cells/well were seeded in each well of 96-well plate. 100 μL of cell culture medium with or without carnosol was added into the well, and the plates were incubated at 37 °C and 5% CO_2_ for 10 days. For harvest, 50 μL agar solubilization solution was added to each well and incubated for 60 minutes at 37 °C. Then 25 μL lysis buffer was added and the fluorescent was read at Ex/Em 485/520 nm.

### Western blot analysis

Cells were lysed with Nonidet P-40 (NP-40) lysis buffer (2% NP-40, 80 mM NaCl, 100 mM Tris-HCl pH 8.0, 0.1% SDS) with proteinase inhibitor mixture (Complete^TM^, Roche Molecular Biochemicals) at indicated time point. Cell lysates were incubated on ice for 15 minutes and then centrifuged at 13,000 rpm at 4 °C for 15 minutes. Protein concentration was measured by Protein DC Assay kit (Bio-Rad Laboratories). Equal amounts of protein were subjected on SDS-PAGE and transferred to nitrocellulose membrane. The membrane was blocked in 5% milk in Tris buffered saline plus Tween 20 (TBST) for 45 minutes and probed with anti-γH2AX (Cell Signaling), anti-pCHK1 (Cell Signaling), anti-p276-NF-κB (Santa Cruz), anti-NF-κB p65 (Santa Cruz), anti-IκB (Santa Cruz), or anti-β-actin (Santa Cruz) at 4 °C overnight. After washing with TBST, the membrane was incubated with corresponding HRP-conjugated anti-rabbit or anti-mouse antibody for 45 minutes at room temperature. Membrane was then washed three times in TBST, followed by two times wash in TBS and developed in West Pico Supersignal chemiluminescent substrate (Pierce).

### Immunofluorescence staining of phosphorylated Chk1

Cells were fixed with 3.6% formaldehyde for 10 minutes at room temperature, rinsed with PBS three times and permeabilized with 0.1% Triton X-100 in PBS for 5 minutes. Cells were then blocked with blocking buffer (2 mg/mL BSA in PBS) for 60 minutes before incubating with anti-p-Chk1 antibody (Cell Signaling) for 60 minutes. After three times washing with PBS, cells were incubated with a fluorescein-conjugated horse anti-rabbit antibody (Vector Labs) for 60 minutes, washed with PBS and mounted with ProLong Gold Antifade Reagent with 4′,6-diamidino-2-phenylindole (DAPI) (Invitrogen). The pictures were taken by NIKON Eclipse E600.

### Comet Assay

HaCaT cells (with or without carnosol treatment) were collected 15 minutes after 50 mJ/cm^2^ UVB radiation. The alkaline comet assay was performed according to manufacturer’s instructions (Trevigen). LMAgarose was added to the cells then pipetted onto CometSlide. After the solidification of the agarose, the slides were immersed into lysis solution for 60 minutes at 4 °C and transferred to alkaline unwinding solution for 60 minutes at 4 °C. The slides were then run in an electrophoresis tank with alkaline electrophoresis solution at 21 volts for 40 minutes, immersed twice in dH_2_O for 5 minutes each, and then in 70% ethanol for 5 minutes. After that, slides were dried at 37 °C for 15 minutes and stained with SYBR gold and detected by fluorescent microscopy at Ex/Em 496/540 nm.

### CPD and 6–4 PP ELISA assay

HaCaT cells (with or without carnosol treatment) were collected 15 minutes after 50 mJ/cm^2^ UVB radiation. CPD and 6–4 PP detection assays were performed following manufacturer’s instructions (Cell Biolabs Inc.). DNA was extracted from the cells (Qiagen), heated at 95 °C for 10 minutes and chilled on ice for 10 minutes. Then, 50 μL of 4 μg/mL DNA was added in each well with 50 μL DNA binding solution and incubated overnight. The solution was removed the next day and the wells were washed twice with PBS before adding anti-CPD or anti-6–4 PP antibody. After 60 minutes incubation at room temperature, the wells were washed 5 times with washing buffer followed by 60 minutes incubation with 100 μL secondary antibody. 100 μL substrate solution was then added and incubated for 15 minutes, stopped by adding 100 μL stop solution. Results were read at OD450 nm.

### Electrophoretic mobility shift assay

A 22-bp synthetic oligonucleotide (5′-AGTTGAGGGGACTTTCCCAGGC-3′) containing the specific NF-κB-binding site was annealed and labeled with γ- ^32^P-ATP using T4 polynucleotide kinase. A DNA-binding reaction mixture of total 20 μL containing poly (dI:dC), labeled probe, binding buffer (10 mM pH 8.0 Tris HCl, 150 mM KCl, 0.5 mM EDTA, 0.1% Triton-X 100, 12.5% Glycerol and 0.2 mM DTT) and 10 μg of cell nuclear extract was incubated at room temperature for 30 minutes and loaded onto a 5% non-denaturing polyacrylamide gel for electrophoresis. The gel was run in 0.5 × TBE buffer at 120 V, transferred to a double layer of Whatman paper and dried on a gel dryer for 45–60 minutes at 76 °C. The dried gel was exposed to autoradiography film (Denville) at −80 °C, the NF-κB bound ^32^P-labeled DNA was detected and the band intensity was analyzed by Image J.

### Cell survival analysis

Total cell number of 1 × 10^5^ was used for each analysis using flow cytometry. Fluorescein isothiocyanate (FITC) conjugated-annexin V (ANX5)/propidium iodide (PI) apoptosis detection kit (BD Biosciences) were used to stain the cells through the determination of the loss of membrane phospholipid symmetry and membrane integrity. Cell survival rate (R) was calculated as: R = [1 × 10^5^ – number of positive stained cells]/1 × 10^5^. Both floating and attached cells were harvested and washed twice with cold PBS. The cells were then suspended in ANX5 binding buffer (10 mM Hepes/NaOH, pH 7.4, 140 mM NaCl and 2.5 mM CaCl_2_). The cell suspension was mixed with 5 μL ANX5-FITC and 5 μL PI and incubated for 15 minutes in dark at room temperature. The ANX5/PI double-stained cells were analyzed using a FACSort Flow Cytometer (BD Science) equipped with CellQuest software (BD Science).

### Statistical analysis

Results are expressed as mean ± standard deviation (SD) for three independent experiments. Data were compared between groups using student t-test, *p* < 0.05 was considered as statistically different.

## Electronic supplementary material

Supplementary Information
