# Supplementary material for: The Mechanisms of Carnosol in Chemoprevention of Ultraviolet B-Light-Induced Non-Melanoma Skin Cancer Formation
Source: Sci Rep. 2018 Feb 23;8:3574. doi: 10.1038/s41598-018-22029-x (PMC5824785; doi:10.1038/s41598-018-22029-x)
Supplement: Supplementary file 1 — Supplementary Information [file 41598_2018_22029_MOESM1_ESM.pdf]

Title:

The Mechanisms of Carnosol in Chemoprevention of Ultraviolet B-Light-Induced Non-melanoma Skin Cancer Formation

Authors:
Lingying Tong
Shiyong Wu

Fig 3A

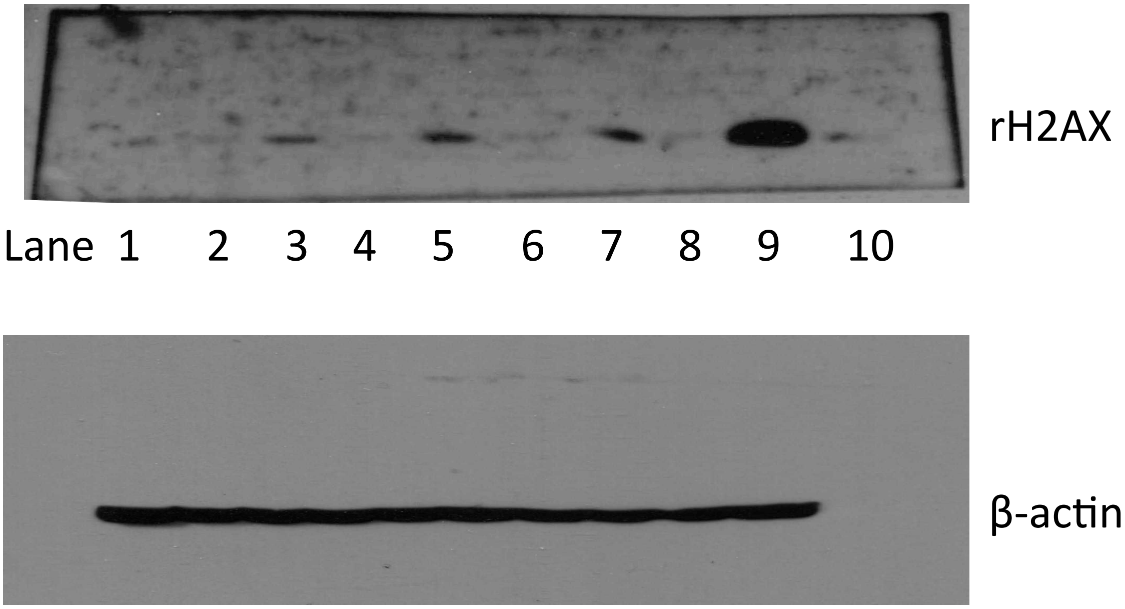

Western blot analysis for rH2AX and β-actin. Lane 1 and 2 HaCaT cell samples with and without carnosol treatment. Lane 3 and 4 : HaCaT cell samples collected at 5 min post UVB radiation, with and without carnosol treatment. Lane 5 and 6: : HaCaT cell samples collected at 10 min post UVB radiation, with and without carnosol treatment. Lane 7 and 8: HaCaT cell samples collected at 15 min post UVB radiation, with and without carnosol treatment. Lane 9 and 10: HaCaT cell samples collected at 60 min post UVB radiation, with and without carnosol treatment.

Fig 3A

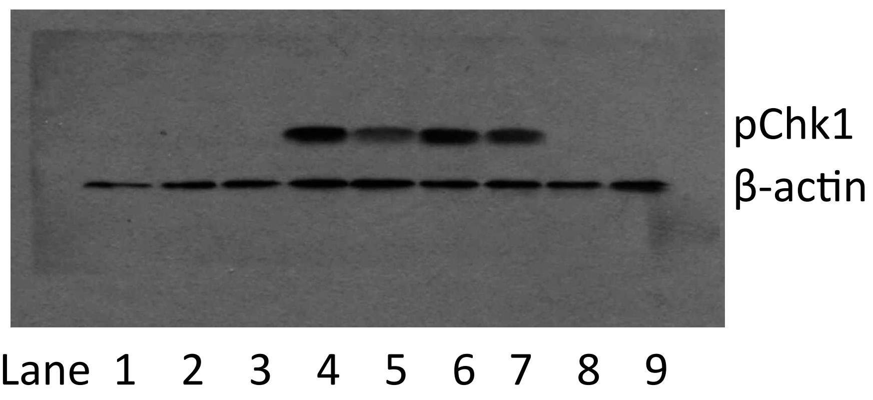

Western blot analysis for pChk1 and β-actin. Lane 1 Mouse embryonic fibroblast cell samples (irrelevant to the manuscript). Lane 2 and HaCaT cell samples with and without carnosol treatment. Lane 4 and 5: HaCaT cell samples collected at 15 min post UVB radiation, with and without carnosol treatment. Lane 6 and 7: HaCaT cell samples collected at 60 min post UVB radiation, with and without carnosol treatment. Lane 8 and 9: HaCaT cell samples collected at 12 h post UVB radiation, with and without carnosol treatment.

Fig 7A

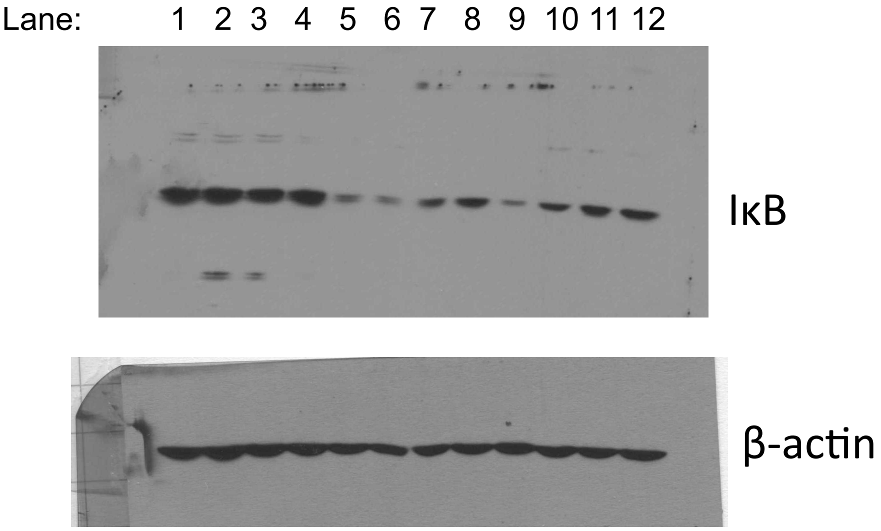

Western blot analysis for IκB and β-actin. Lane 1-4: Cells treated with carnosol for 0, 1, 10, 20 uM. Lane 5-8: Cells were treated with 0, 1, 10, 20 uM carnosol, and collected at 2 h post UVB radiation. Lane 9-12: Cells were treated with 0, 1, 10, 20 uM carnosol, and collected at 4 h post UVB radiation.

Fig 7B

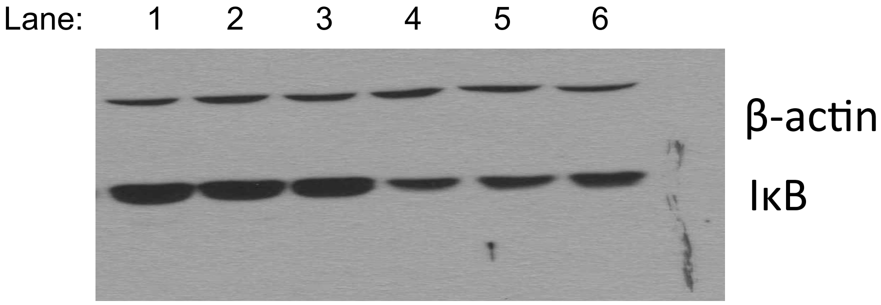

Western blot analysis for IκB and β-actin. Lane 1: HaCaT control; Lane 2: 10 μM carnosol treatment; Lane 3: 20 μM carnosol treatment; Lane 4: UVB radiation; Lane 5: UVB radiation with 10 μM carnosol treatment; Lane 6: UVB radiation with 20 μM carnosol treatment.

Fig 7C

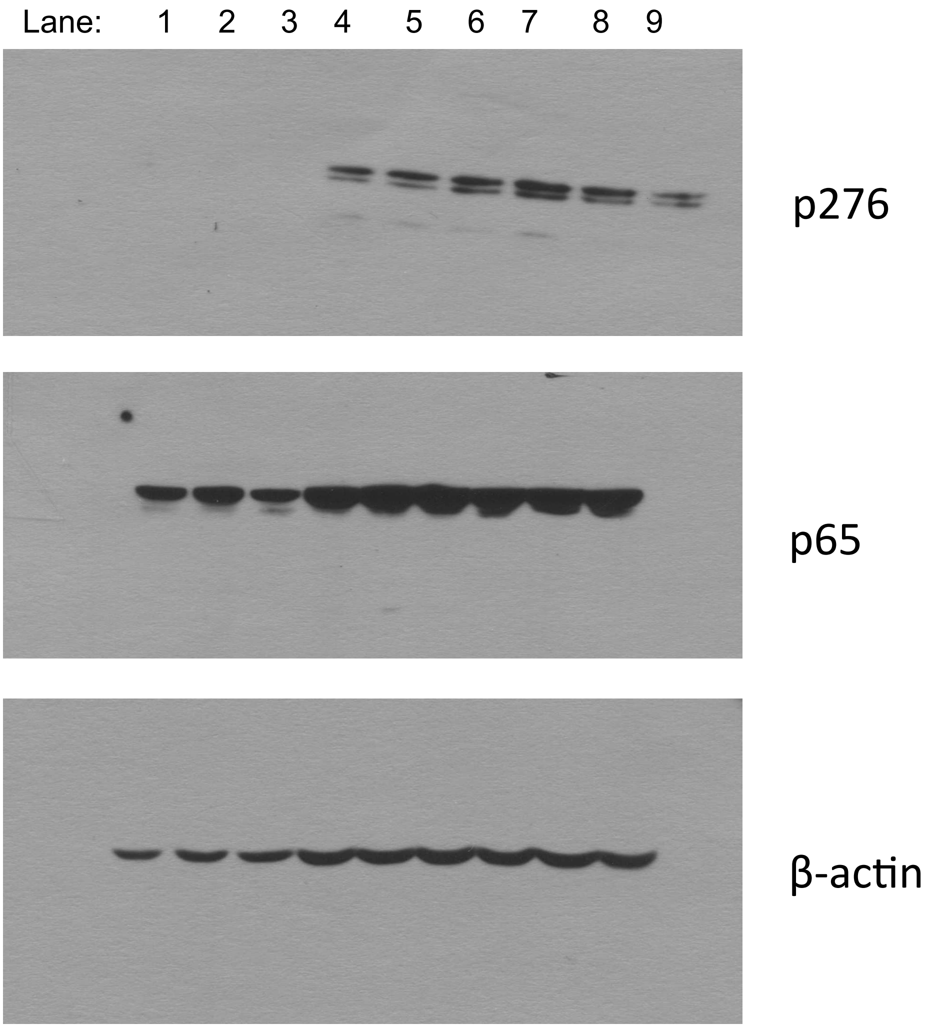

Western blot analysis for p276 (phosphorylation of NF-κB), p65 (total NF-κB) and β-actin. Lanes 1-3: control HaCaT cells treated with 0, 10, 20 µM carnosol; Lane 4-6: HaCaT cells radiated with 5 mJ/cm^2 UVB radiation with 0, 10, 20 µM carnosol treatment; Lane 7-9: HaCaT cells radiated with 50 mJ/cm^2 UVB radiation with 0, 10, 20 µM carnosol.

Fig 7D

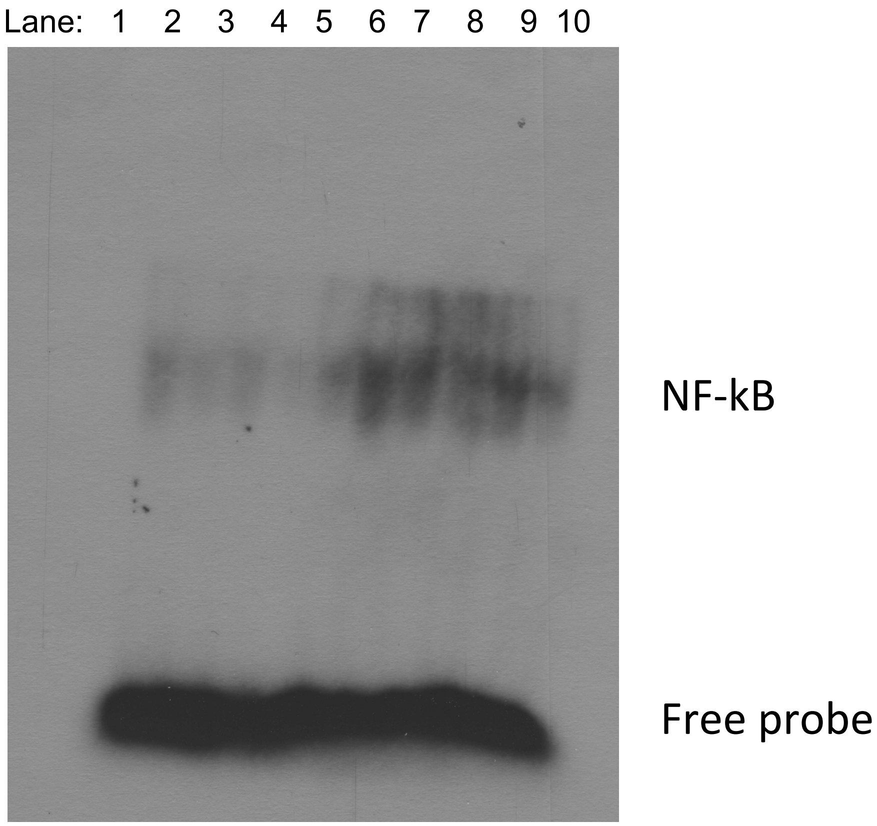

EMSA blot for NF-κB activity. HaCaT cells were treated with carnosol for 2, 4, 6 hours after UVB radiation. Lane 1-4: cells treated with carnosol for 0, 2, 4, 6h. Lane 5-7: Cells treated and collected at 2, 4, 6 h post UVB radiation. Lane 8-10: Cells were treated with carnosol and UVB radiation, and cells were collect at 2, 4, 6 h post UVB radiation.
